# Massive pericardial effusion as the first manifestation of childhood non‐Hodgkin's lymphoma: A case report

**DOI:** 10.1002/ccr3.5105

**Published:** 2021-11-25

**Authors:** Behzad Alizadeh, Zahra Shaye, Zahra Badiea, Paria Dehghanian

**Affiliations:** ^1^ Faculty of Medicine Interventional Pediatric Cardiology, Pediatric and Congenital Cardiology Division Pediatric Department Imam Reza Training Hospital Mashhad University of Medical Sciences Mashhad Iran; ^2^ Mashhad University of Medical Sciences Mashhad Iran; ^3^ Pediatric Hematology and Oncology Mashhad University of Medical Sciences Mashhad Iran; ^4^ Pediatric Pathologist Mashhad University of Medical Sciences Mashhad Iran

**Keywords:** case report, non‐Hodgkin lymphoma, pediatrics, pericardial effusion

## Abstract

Although one of the serious manifestations of advanced malignancies is pericardial involvement, pericardial involvement of lymphoma is extremely rare. We present a case of a 6‐year‐old girl arriving at the hospital with dyspnea and pleuritic chest pain, which is eventually diagnosed with massive pericardial effusion due to mediastinal non‐Hodgkin lymphoma.

## Graphical abstract

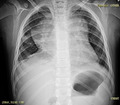

## 1 INTRODUCTION

One of the important causes of pericardial effusion are solid tumors.^1^ Pericardial involvement from non‐Hodgkin lymphoma is uncommon and malignant pericardial effusion is even more scarce.^2^ Pericardial effusion, which is the accumulation of fluid in the pericardial sac, is commonly associated with significant decrease in patient survival and may lead to grave consequences such as tamponade, cardiovascular collapse, and death.^3^, ^4^ Here, we present a case of this complication as the first manifestation of mediastinal lymphoma.

## 2 CASE

A 6‐year‐old girl presented to the hospital with the main complaint of dyspnea and pleuritic chest pain starting from 1 week earlier. She was mildly agitated and could not lie down due to pain and orthopnea; She was also febrile during this period. In her past medical history, she is the first sibling, was born term, with no previous hospitalization. Her parents reported intermittent fever for 3 months before admission. There was no close contact with a suspicious COVID‐19 patient.

Upon physical examination, general appearance was good and the child was mentally oriented. Lung auscultation was clear but a region of decreased sounds was detected on the lower right side, heart sounds were muffled, and the peripheral pulses were normal at the time. The blood pressure reading was 100/65, heart rate was 120, and respiratory rate was 30. Abdominal examination revealed hepatomegaly.

**FIGURE 1:**
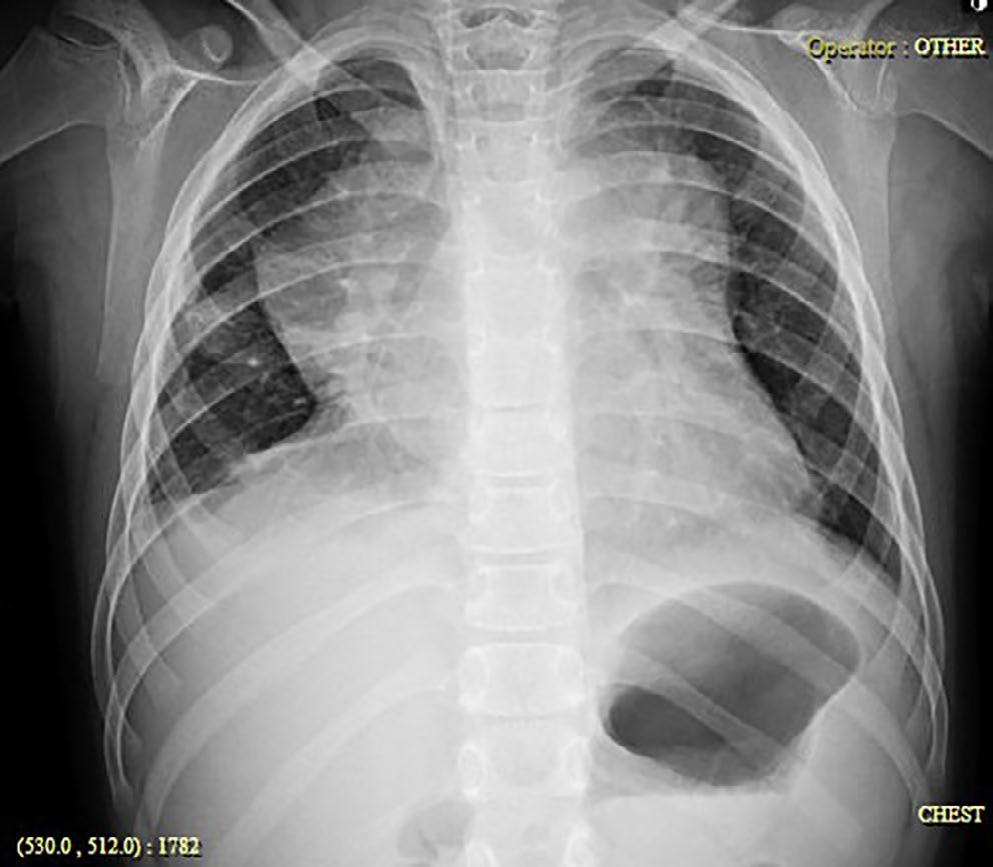
Patient's chest radiograph demonstrating a mediastinal widening, suggestive of mediastinal mass

**FIGURE 2:**
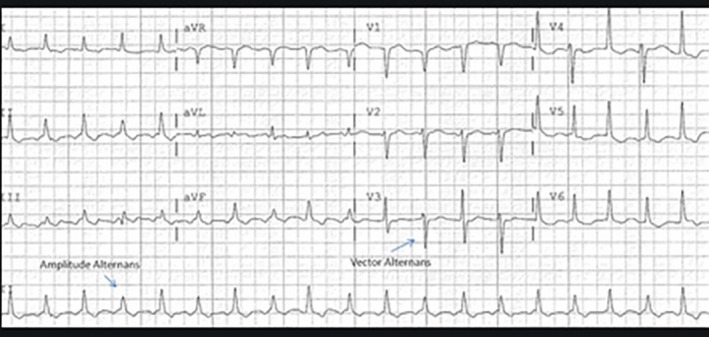
Changing QRS complex amplitude on a 12‐lead electrocardiogram: Electrical alternans

Initial paraclinical studies included chest radiograph which showed a mediastinal widening (Figure 1) and 12‐lead electrocardiogram revealed sinus tachycardia and low‐voltage alternans QRS (Figure 2). Complete blood test, blood biochemistry assessment, and COVID‐19 PCR test were all normal (Table 1).

**TABLE 1:** Complete blood count and biochemical tests

White blood cells count	11.4 (PMN69, LYMPH21)	Blood sugar	150
Hemoglobin	12.6	UREA	25
Hematocrit	36.1	Creatinine	0.6
Mean corpuscular volume	71.9	LDH	1061
Mean corpuscular hemoglobin	25	Sodium	139
Mean corpuscular hemoglobin concentration	34.9	Potassium	4.7
Platelet count	565	Troponin I	Negative
		Uric Acid	4.5
Covid19	Negative	Calcium	10
C‐reactive protein	66	Phosphorus	4
Erythrocyte sedimentation rate	57	Alanine transaminase	27
		Aspartate transaminase	34

**FIGURE 3:**
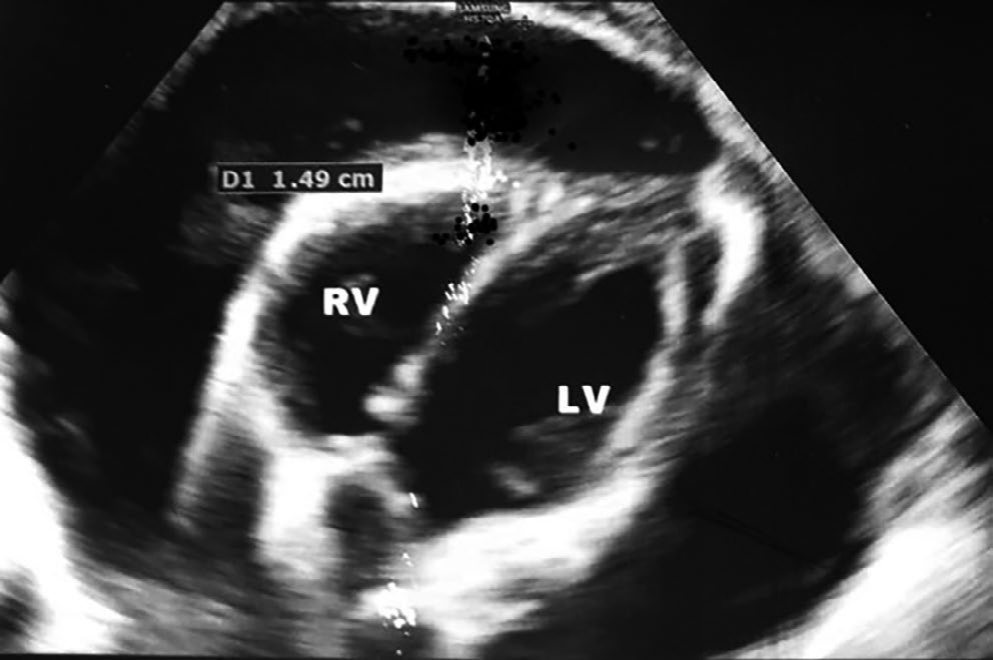
Transthoracic echocardiogram. The heart is seen surrounded by a very large pericardial effusion, without any sign of tamponade sign (15 mm left side & 18 mm right side)

**FIGURE 4:**
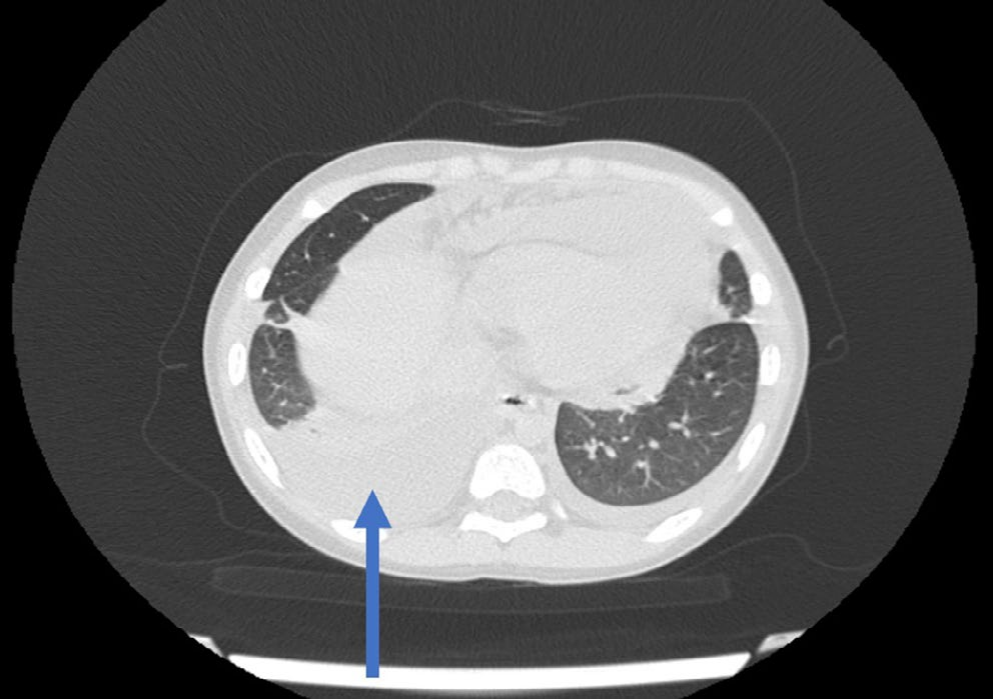
Thorax computed tomography (CT) scan revealed a mediastinal mass accompanied by pleural and pericardial effusion (arrows)

Further evaluations were performed based on the primary findings, which included echocardiography showing significant pericardial effusion, and thorax computed tomography (CT) scan revealing a mediastinal mass accompanied by pleural and pericardial effusion (Figures 3 and 4).

**FIGURE 5:**
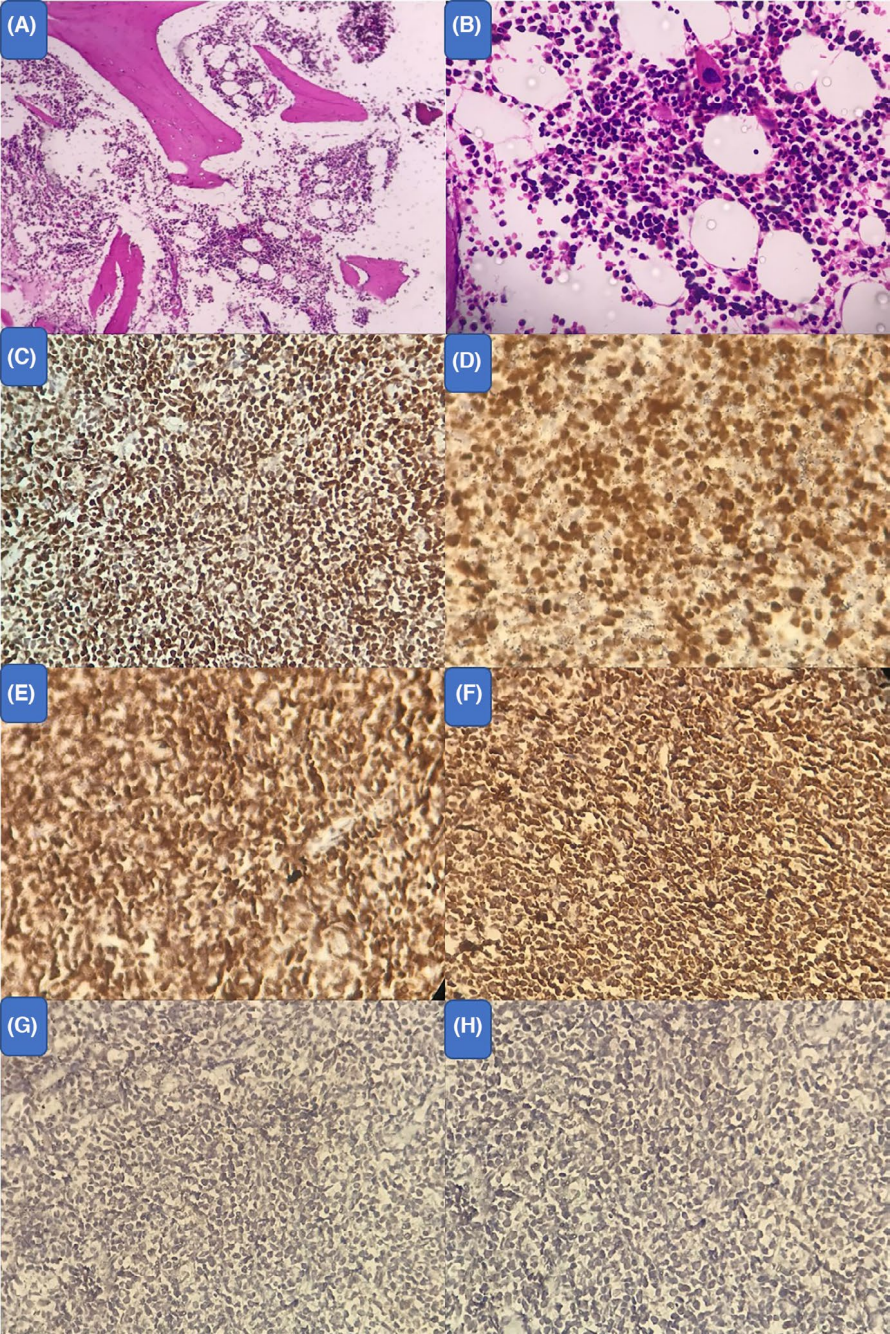
Morphologic features and IHC study results are consistent with T‐cell lymphoblastic lymphoma: Cellular hematopoietic mass, non‐neoplastic (H&E staining): Low power field (A) and high‐power field (B). (C) Positive CD99 IHC staining of tumor cells. (D) Positive ki‐67 IHC staining in more than 90%of tumor cells. E) Positive TDT IHC staining of tumor cells. (F) Positive CD3 IHC staining of tumor cells. (G) Negative CD10 IHC staining of tumor cells. (H) Negative CD20 IHC staining of tumor cells

To rule out thymoma, lymphoma and also germ cell tumors, a core needle biopsy (CNB) from the mass was obtained, which was in favor of mediastinal non‐Hodgkin lymphoma (malignant small round cell tumor), although the bone marrow aspiration and biopsy were normal. The morphologic features and IHC study results were consistent with T‐cell lymphoblastic lymphoma (Figure 5). Chemotherapy was initiated after referral to oncologist, immunohistochemistry tests, and complete staging. The chemotherapy regimen was consisted from Prednisone (oral administration), vincristine (intravenous administration on), daunorubicin (intravenous administration), asparaginase (intravenous administration), cyclophosphamide (intravenous administration), cytarabine (intravenous administration), mercaptopurine (oral administration), and methotrexate (intrathecal administration). After 1 year of follow‐up, the patient is disease free.

## 3 DISCUSSION

One of the frequent and serious manifestations of advanced malignancies is pericardial involvement, which includes various degrees such as pericarditis, pericardial effusion, pericardial tamponade, and constrictive pericarditis.^4^ Symptomatic effusion mostly presents with tachycardia, dyspnea (our patient's chief complaint), and echocardiographic characteristics of right heart failure, but it can also occur asymptomatically.^5^, ^6^ It needs special attention particularly during the Era of the coronavirus (COVID‐19) pandemic, which most of these symptoms mimic the SARS‐CoV‐2 infection and also suffering from COVID‐19 may alter the prognosis of patients with malignancies,^7^, ^8^ and those patients need special attention.^9^ Pericardial effusion rises mainly from blockage of the lymphatic and venous drainage of the pericardium, which is caused by adjacent compression, direct tumoral infiltration, or via hematogenous spread. Pericardial effusions in cancer patients can also be triggered by chemotherapy and radiotherapy. Other complications, such as infections and autoimmune diseases, can also cause pericardial effusion in these patients.^4^, ^10^ The typical findings on electrocardiogram are sinus tachycardia and low voltage defined as maximum QRS amplitude <0.5 mV in the limb leads. New‐onset atrial fibrillation can be present. Electrical alternans, characterized by beat‐to‐beat alternation in the QRS complex, represent the swinging of the heart in the pericardial fluid.^3^ Echocardiography is considered to be the primary imaging modality for pericardial effusion, and it is recommended to be done before, during, and after treatment to monitor the patient.^1^, ^2^

Lymphoma is a hematological malignancy that emerges from the clonal proliferation of lymphocytes at different maturation stages. It presents with fatigue, night sweats, enlarged painless lymph nodes, and weight loss. Lymphoma is classified into two major subgroups: Hodgkin (10%) and non‐Hodgkin lymphoma (90%).^2^ Lymphomas involving the mediastinum have a wide age range and occur in both pediatric and adult populations.^11^

Chen et al compared pediatric and adult lymphomas involving the mediastinum and reported that pediatric patients had a higher incidence of T‐LBL/T‐ALL, prevalence of dyspnea, stage IV tumors frequency, and relative tumor diameter, compared to adults. They were also more likely to be male.^12^

Cardiac and pericardial involvement of lymphoma is extremely rare. It accounts for 0.5% of cardiac involvement and 1% of all extranodal non‐Hodgkin lymphomas. It is more common in high‐grade lymphomas, particularly double‐hit/triple‐hit subtypes, and shows a poor prognosis, as in other malignancies.^6^ Bertog et al. studied 163 patients diagnosed with constrictive pericarditis, and lymphoma was the etiology in only two of the patients.^13^ In another study, out of eight patients undergoing pericardiectomy for malignant constrictive pericarditis, only one was diagnosed with lymphoma.^14^ This unlikeliness has sometimes led to incorrect approaches and treating patients with multiple pericardiocenteses or management according to other diagnoses such as tuberculosis.^15^

On the contrary, malignancy involving the pericardium is usually a late secondary feature.^16^ In a case study‐based systematic review on lymphoma‐associated cardiac tamponade, Shareef et al^4^ evaluated 52 cases aged 9–95 (median 52), out of which 49 patients had non‐Hodgkin lymphoma, and observed that most of these patients were diagnosed with lymphoma prior to hospital presentation (80.8%). They also reported that the median overall survival of patients with lymphoma and cardiac tamponade is 4 months, and there is no significant difference between lymphoma diagnosis before or after this complication.

A chest x‐ray identifies an anterior mediastinal mass mostly when it is very large (bulky disease) and produces mediastinal widening. It demonstrates tumor bulk and pleural effusion. Chest computed tomography (CT) scans are examined for precise tumor location, presence of necrosis, pulmonary parenchymal involvement, chest wall invasion, and more assessment of pleuropericardial effusion.^17^, ^18^ Definitive diagnosis is achieved via biopsy and histopathological examination, and treatment begins after complete staging according to Ann Arbor classification.

Currently, there is an emerging evidence on the underlying molecular mechanisms of malignancies, which may improve the outcome of patients through introducing new drugs targeting tumor‐specific pathways.^19^, ^20^, ^21^, ^22^, ^23^ Besides, new radiotherapy techniques can be considered to treat tumoral lesions while decreasing the irradiated volume of organ at risks.^24^, ^25^

## 4 CONCLUSION

Although pericardial effusion is rare as the first demonstration of pediatric mediastinal lymphoma, it is important to take this differential diagnosis into consideration. Because non‐Hodgkin lymphomas with mediastinal disease at presentation have a natural tendency for rapid dissemination and worse prognosis when invading the pericardium; hence, minimal delay in diagnosis is essential to avoid incorrect initial management and initiate the true approach as soon as possible.

## CONFLICT OF INTEREST

All authors declare that they have no conflict of interest.

## AUTHOR CONTRIBUTIONS

B.A., Z.B., Z. Sh., and P.D. contributed in conception, design, and drafting of the manuscript. Z. Sh. contributed in data collection. B.A., Z.B., and Z. Sh. contributed in drafting of the manuscript and P.D. supervised the study. All authors approved the final version for submission.

## ETHICAL APPROVAL

The study protocol was approved by the Ethics Committee of Mashhad University of Medical Sciences and was conducted according to the Declaration of Helsinki.

## CONSENT

Undersigned informed consent form was obtained from patient prior to the enrollment.

## Data Availability

The data sets used and/or analyzed during the current study are available from the corresponding authors per request.
